# Genetic basis of wing morphogenesis in *Drosophila*: sexual dimorphism and non-allometric effects of shape variation

**DOI:** 10.1186/1471-213X-11-32

**Published:** 2011-06-02

**Authors:** Valeria P Carreira, Ignacio M Soto, Julián Mensch, Juan J Fanara

**Affiliations:** 1 Departamento de Ecología, Genética y Evolución. Facultad de Ciencias Exactas y Naturales. Universidad de Buenos Aires. Ciudad Universitaria, Pabellón II (C1428 EHA) Buenos Aires. Argentina

## Abstract

**Background:**

The *Drosophila *wing represents a particularly appropriate model to investigate the developmental control of phenotypic variation. Previous studies which aimed to identify candidate genes for wing morphology demonstrated that the genetic basis of wing shape variation in *D. melanogaster *is composed of numerous genetic factors causing small, additive effects. In this study, we analyzed wing shape in males and females from 191 lines of *D. melanogaster*, homozygous for a single *P*-element insertion, using geometric morphometrics techniques. The analysis allowed us to identify known and novel candidate genes that may contribute to the expression of wing shape in each sex separately and to compare them to candidate genes affecting wing size which have been identified previously using the same lines.

**Results:**

Our results indicate that more than 63% of induced mutations affected wing shape in one or both sexes, although only 33% showed significant differences in both males and females. The joint analysis of wing size and shape revealed that only 19% of the *P*-element insertions caused coincident effects on both components of wing form in one or both sexes. Further morphometrical analyses revealed that the intersection between veins showed the smallest displacements in the proximal region of the wing. Finally, we observed that mutations causing general deformations were more common than expected in both sexes whereas the opposite occurred with those generating local changes. For most of the 94 candidate genes identified, this seems to be the first record relating them with wing shape variation.

**Conclusions:**

Our results support the idea that the genetic architecture of wing shape is complex with many different genes contributing to the trait in a sexually dimorphic manner. This polygenic basis, which is relatively independent from that of wing size, is composed of genes generally involved in development and/or metabolic functions, especially related to the regulation of different cellular processes such as motility, adhesion, communication and signal transduction. This study suggests that understanding the genetic basis of wing shape requires merging the regulation of vein patterning by signalling pathways with processes that occur during wing development at the cellular level.

## Background

In general, organ development is organized in two parts; the first is related to the generation of positional information across a field of cells and the second to the refinement of mature form (i.e. organ's size and shape) [1]. The *Drosophila *wing represents a particularly appropriate model to investigate the developmental control of phenotypic variation. It is involved in several functions of ecological and evolutionary importance (e.g., flight and male courtship song) and its developmental genetics is extensively understood [2]. Molecular genetic dissection of *Drosophila *wing development has revealed how the dorsal-ventral and anterior-posterior axes are established and how morphogens emanating from these organizing regions establish the positions of the future veins, hence providing a general description of wing pattern formation (see [3] for a complete review of the subject). In this process, veins that have been specified may be lost later in development due to unsuccessful maintenance and early-appearing ectopic venation may be lost or reduced by subsequent refinement [3]. In this respect, the ancestor of extant *Drosophila *probably had certain mechanisms that specified additional veins (reviewed in [4]). Furthermore, *Drosophila *may retain some of this information as the ectopic veins produced after genetic manipulations could eventually imitate aspects of the ancestral pattern [5,6]. Therefore, if veins have been fused during evolution, formation of a single *Drosophila *vein may depend on a combination of mechanisms that determined different veins in its ancestors [3]. Even though vein patterning is highly conserved among *Drosophila *species, researchers have isolated numerous mutations affecting vein formation (reviewed in [7,8]). Many of the mutated loci encode components of known intercellular signaling pathways. There is evidence that five of them contribute to vein position and maintain vein and intervein territories in the wing: Hedgehog signaling, bone morphogenetic protein signaling, *Drosophila *epidermal growth factor receptor signaling, signaling mediated by Wingless (Wg), and Notch signaling mediated by its ligands, Delta and Serrate [3]. In addition, components belonging to different signaling pathways seem to interact, forming networks which establish vein and intervein regions [9-11].

**Figure 1:**
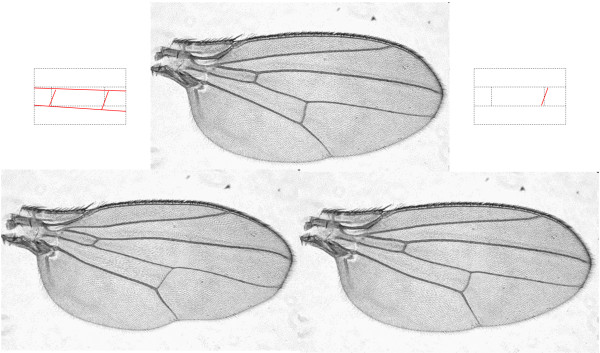
**Global versus local wing shape changes**. General (on the left) and localized (on the right) deformations of normal wing shape (central image) which might be studied using geometric morphometrics. Each type of change is illustrated by a picture (at the bottom) and a diagram (above the corresponding image) which shows the modifications in red.

Developmental processes such as wing patterning have been mostly investigated following classical Mendelian and molecular genetic methodologies [12]. However, since it presents a geometric conformation, changes in vein disposition might be studied using geometric morphometrics techniques. This would allow for the analysis of shape modifications at different spatial scales (i.e. general versus localized deformations; Figure 1) independent of wing size changes. These morphological differences may be caused by genetic and/or environmental alterations which could be identified using quantitative genetics approaches. Previous quantitative and evolutionary genetic analyses have mainly used QTL and linkage disequilibrium mapping to identify candidate genes for these characters, but large-scale screenings using mutagenesis have also been employed. Quantitative genetic analysis of the subtle effects of *P*-element mutations that have been induced in an isogenic background [13,14] is a highly efficient method for functional genomic studies [15-21].

Many studies have demonstrated that wing shape in *D. melanogaster *exhibits moderate to high heritability [22-26]. They have also shown that numerous genetic factors affect wing shape variation via small, additive effects [22,26-31]. These results agree with those obtained by Mezey and Houle [24] who estimated the minimum number of dimensions (considering wing shape as a multivariate trait) in which there is additive variance as 20, implying that the number of genes with an additive affect on wing shape should be at least 20. According to Weber and collaborators [32], even though novel mutations generated in the laboratory may not contribute to evolutionary change as much as natural alleles, the study of their phenotypic effects would help to elucidate the genetic architecture of wing shape (i.e. it might reveal the existence of selective constraints on wing shape). In this sense, their study demonstrated that at least 11 of 50 random *P*-element insertion lines had a replicable, significant effect on this trait in an isogenic background [32]. These mutations affected 13 candidate genes which are involved in different developmental and physiological processes [32]. Although the method used to examine shape in this study (four univariate measures of allometry) probably underestimated phenotypic variation, the results obtained by Weber and collaborators are consistent with a large mutational target for wing shape. Dworkin and Gibson [12] then addressed the potential role of genes belonging to signaling pathways mentioned above with respect to wing shape in *D. melanogaster*. With this aim, they analyzed the wings corresponding to 50 *P*-element insertion lines using more potent techniques derived from geometric morphometrics [12]. They showed that most of the mutations had significant effects on wing shape without affecting wing size [12]. Furthermore, they demonstrated that the effects of mutations do not group according to the signaling pathways to which the genes belong [12], supporting the existence of interactions between pathways [3].

In this study, we analyzed wing shape in males and females from 191 *P*-element insertion lines using geometric morphometrics techniques. Since these lines have the same genetic background, they only differ in the exact position of the *P*-element. Therefore, as all lines studied were raised under controlled environmental conditions, the analyses allowed us to associate phenotypic variation primarily to its genetic causes. In particular, we could identify candidate genes that contribute to the expression of wing shape in each sex separately and to compare them to candidate genes affecting wing size which have been previously identified using the same 191 lines [16]. In addition, we classified the lines according to the spatial scale of geometric changes, which enabled us to study the occurrence of mutants showing global shape changes in comparison with those that presented local phenotypic effects. Our results indicate that the genetic architecture of wing shape involves a large fraction of the genome and it is largely sex specific and independent of wing size. Functional analysis of the 94 candidate genes identified revealed that the regulation of cell adhesion and cell motility, cell communication and signal transduction are important biological processes involved in wing morphogenesis.

## Results

### Identification of divergent lines for wing shape and simultaneous analysis of both components of wing form

The results of the MANOVAs indicate that a large number of transposon-tagged genes affect the expression of wing shape since 63.4% of the lines (121 out of 191 lines analyzed) showed significant differences from the control line in at least one sex (See additional file 1: Mutant lines used with the respective candidate genes). Nearly half of the lines (49.7% in males and 46.6% in females) showed significant differences from the control for wing shape when data were analyzed for each sex separately (See additional file 1). However, only 33% of the lines (63 out of 191 lines studied) showed significant differences in both sexes (See additional file 1).

**Figure 2:**
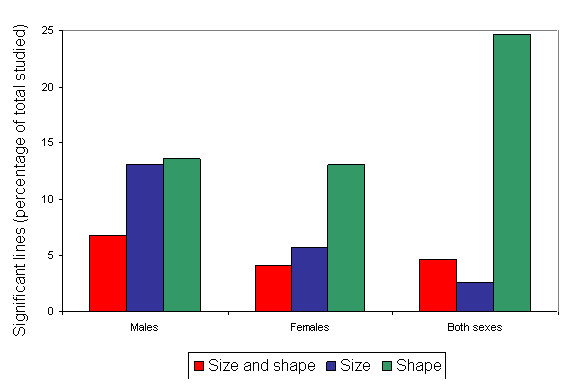
**Mutations affecting wing size and/or shape in one or both sexes**. Percentage of significant lines with respect to the total studied in which *P*-element insertions affected wing form in a specific way (size and/or shape) in each sex and in both sexes similarly. Significant lines that did not show a similar effect in both sexes were distributed between sexes in a non-exclusive manner (that is, a given line might be associated to one or both sexes).

Analysis of both components of wing form (wing size and shape) indicate that 150 out of 191 lines analyzed (78.5%) showed significant differences from the control line for at least one of the traits in either sex (See additional file 1). More than half of the lines (65.4% in males and 55.0% in females) showed those differences when sexes were analyzed separately (See additional file 1). However, only 19.4% of the lines (37 out of 191 lines studied) showed the mentioned differences for both traits in one or both sexes (See additional file 1). This percentage diminishes by approximately one half (11.5% in males and 8.9% in females) when data were analyzed for each sex separately (See additional file 1). The percentage of lines in which the *P*-element insertion affected only wing shape (44.0%) was larger than that corresponding to the lines wherein mutations modified only wing size (15.2%; See additional file 1). This relationship was maintained when the data were analyzed for each sex separately (38.2% versus 15.7% in males and 37.7% versus 8.4% in females, for wing shape and wing size respectively; See additional file 1). Frequency analyses showed that the number of cases in which *P*-element insertions affected only wing size, only wing shape or both traits are within the expected values according to an independent behaviour of those traits in males (χ^2^_1 _= 1.578; p = 0.209) as well as in females (χ^2^_1 _= 0.388; p = 0.533). Figure 2 shows the number of lines showing sex specific effects or similar effects in both sexes which were classified according to the phenotypic effect shown (size specific, shape specific or similar for both wing components). This classification required the study of each line separately, which allowed us to note that, only in 61 cases, mutations caused a similar effect (affecting one and/or the other trait) in both sexes (See additional file 1; Figure 2). The rest of the significant lines showed different wing form changes in each sex, with the number of significant lines greater for males than for females (64 and 44 respectively; See additional file 1; Figure 2).

### Gene identification and functional analysis

We identified 94 candidate genes that affect wing shape based on insertion of the *P*-element within 5 kb from the transcription initiation/finalization site. Only the gene nearest to the insertion was selected as candidate gene, except in those cases in which two genes were closer than 1 kb to the *P*-element insertion site and neither disruption occurred in the gene (See additional file 1). In 5 of the 121 significant lines, the *P*-element insertion was close to two genes (BG00735, BG01007, BG01949, BG02286 and BG02439; See additional file 1).

3 out of the 94 genes identified were affected by the *P*-element insertion in two or more lines (See additional file 1). An interesting observation was that different insertion sites (i.e. different *P*-element insertion lines) in the same gene caused generally different phenotypic effects (See additional file 1), indicating that the precise site of the transposon determines its phenotypic effect, as it was observed previously for body size related traits [16] as well as for other characteristics [33]. Mutation of *Laminin A *(*LanA*) affected only wing shape in both sexes of line BG02113 but it changed both components of wing form in males of lines BG02380 and BG02469 (See additional file 1). The *P*-element insertion affecting *CG30492 *in line BG01354 changed wing shape in both sexes but it affected only wing shape in the females of line BG01990 (See additional file 1). The *P*-element insertion affecting *Smrter *(*Smr*) in line BG02262 changed both components of wing form (size and shape) in both sexes, while it affected both traits only in the males of line BG02219 (See additional file 1). In general, these phenotypic differences due to different *P*-element positions near to the same candidate gene might be due to different levels of gene expression or gene product activity.

Genes with significant effects on wing shape were automatically distributed in gene ontology (GO) terms corresponding to the categories "biological process", "molecular function" and "cellular component" in a non-exclusive manner (i.e. a given gene might be associated to more than one GO term) according to their annotations [34] (Table 1). A large proportion of our candidate genes' products are located in intracellular organelles, display protein binding activity and are related to organ or cell development (Table 1).

**Table 1:** Distribution of candidate genes in GO terms.

GO terms	Genes (%)
Biological process	
Cell development/Organ development	28.1
Nervous system development/Cellular protein metabolic process	21.1
Female gamete generation/R cellular metabolic process	19.3
Cell morphogenesis/Cell surface receptor linked signal transduction/RNA metabolic process	17.5
Anatomical structure formation/Transcription	15.8
Regionalization/Larval or pupal development (sensu Insecta)	12.3
Cell migration/Negative R cellular process	10.5
Cell cycle phase/Intracellular signaling cascade/Cell fate commitment/Metamorphosis/Biopolymer modification/Photoreceptor cell differentiation	8.8
Negative R developmental process/Phosphate metabolic process/DNA metabolic process/Nucleotide metabolic process/Positive R cellular process/Male gamete generation	7.0
Imaginal disc-derived appendage development/Positive R metabolic process/Appendage morphogenesis/Determination of adult life span/Macromolecule biosynthetic process/Homophilic cell adhesion/Establishment of cellular localization/Morphogenesis of an epithelium/R signal transduction/R cell proliferation/Ion transport	5.3
R cell differentiation/Open tracheal system development/Embryonic pattern specification/R biosynthetic process/Olfactory behaviour/R embryonic development/R cell adhesion/Embryonic development ending in birth or egg hatching/R cell cycle/GO:0006791/Vesicle-mediated transport/R transferase A	3.5
	
Molecular function	
Protein binding	75.0
Nucleic acid binding	25.0
Ion binding	17.1
Transferase A/Nucleotide binding	13.2
Hydrolase A	7.9
Receptor A/Transcriptional activator A	4.0
Ion transporter A/RNA polymerase II transcription factor A/Ligase A/GTPase regulator A	2.6
Oxidoreductase A/Lipid transporter A/Lyase A/Enzyme activator A/Channel or pore class transporter A/Small protein conjugating enzyme A/Carbohydrate binding/Carrier A/Transposase A/Helicase A	1.3
	
Cellular component	
Intracellular organelle	63.6
Cytoplasm	31.8
Intrinsic to membrane	25.0
Plasma membrane part	11.4
Basal lamina	4.6

Distribution of annotated candidate genes in gene ontology (GO) terms corresponding to the categories "biological process", "molecular function" and "cellular component" using FatiGO. Genes are distributed in a non-exclusive manner (i.e. a given gene might be associated with more than one GO term). The percentage of genes related to each GO term is shown. R: "Regulation of". A: "Activity".

**Figure 3:**
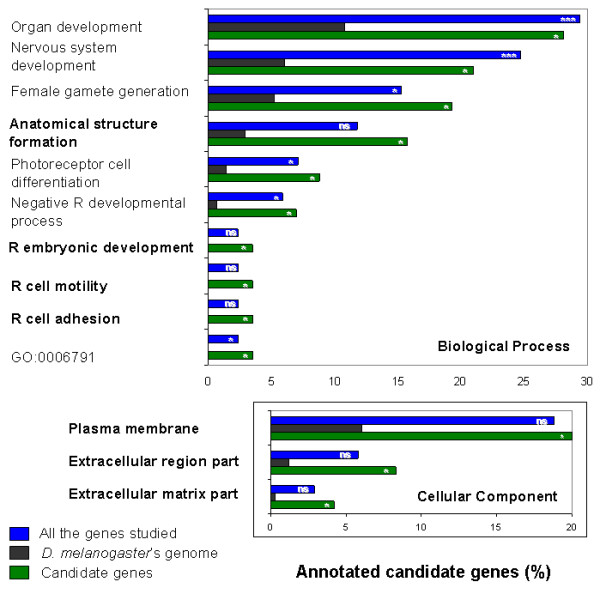
**Functional enrichment analysis of the list of candidate genes for wing shape**. Distribution of annotated genes corresponding to different lists (candidate genes for wing shape, all genes studied and *D. melanogaster*'s genome) in gene ontology (GO) terms belonging to the categories "biological process" and "cellular component". Genes are distributed in a non-exclusive manner (i.e. a given gene might be associated to more than one GO term). ns: not significant; * p < 0.05; *** p < 0.001 (p-values have been adjusted for multiple comparisons). Terms which were over-represented exclusively in the list of candidate genes compared to *D. melanogaster*'s genome and did not show an over-representation in the sample of genes studied are bold-faced. R: "Regulation of".

The distribution of all genes included in the study in GO terms was compared to that of *D. melanogaster*'s genome. Important GO terms were over-represented exclusively in the list of candidate genes compared to *D. melanogaster*'s genome and did not show an over-representation in the list corresponding to all genes studied (Figure 3) which means that the sample studied was not particularly enriched with those GO terms. The terms considered as the most informative are anatomical structure formation, regulation of cell adhesion, regulation of cell motility (corresponding to the category "biological process") and plasma membrane (corresponding to the category "cellular component"; Figure 3).

So far, when a *P*-element insertion was near to a gene in a line showing altered wing morphology, we considered that gene as candidate to be involved in normal wing development. Another possibility is that *P*-element insertions outside of the gene's coding sequence might result in ectopic gene expression in the wing causing morphology changes, even though the candidate gene is not normally involved in wing development. However, results of GO analyses suggest that the majority of our candidate genes would not be false positives as most of the respective terms seem to be associated with development and/or metabolic functions which might be related to morphogenesis.

### Classification of significant lines according to the spatial scale of wing shape deformations

Dunnett contrasts using the scores of the first three relative warps (RWs) corresponding to a value of α = -1 and α = 1 independently (see Methods section), allowed us to separate the significant lines of each sex among four groups according to the spatial scale of wing shape deformations (Table 2). As it is explained below, lines of group I showed general deformations; lines of group II showed localized deformations; lines of group III showed general as well as localized deformations; and lines of group IV showed intermediate deformations. The frequency distribution of the lines among groups showed significant differences between sexes (χ^2^_3 _= 9.853; p = 0.020). The greatest difference between sexes was observed for group III, which was strongly over-represented in males (Table 2). Also, a large difference was observed for group IV which was slightly under-represented in males and slightly over-represented in females (Table 2). These results suggest a sexually dimorphic effect of mutations on wing shape. Consequently, frequency analysis was performed on each sex separately revealing that the number of lines was not proportionally distributed among groups neither in males (χ^2^_3 _= 14.768; p = 0.002) nor in females (χ^2^_3 _= 12.348; p = 0.006; Table 2). The greatest difference between observed and expected values was seen for group II in both sexes, which was under-represented in both cases (Table 2). The other case that showed a similar pattern in both sexes corresponds to group I, which was slightly over-represented in both cases (Table 2). In other words, the number of lines which showed deformations that involved almost the whole wing was greater than expected by chance while the number of lines which showed localized deformations was less than expected.

**Table 2:** Grouping of lines according to the scale of wing shape deformations.

Group	I	II	III	IV
	
Males				
Number of lines	30 (31.6%)	11 (11.6%)	35 (36.8%)	19 (20.0%)
Genes affected by *p[GT1] *insertions	*tal-1A, Hsp 27, inv, nemy, CG42708, msn, mbl, fs(1)h, hdc, Dnr1, l(3)82Fd, CG32572, Fili, l(1)G0007, CG42268, NFAT, dally, Smr *(BG02219, BG02262)*, CG6301, CG14478*	*Trl, fz, CG6767, CG30492 *(BG01354)*, trn, caps*	*CG1678, CG11226, siz, CG10581, Karl, foxo, βInt-ν, boi, Got1, Sip1, Btk29A, Mer, α-Est10, aret, CG42684, chinmo, CG6398, sd, tmod, clt, 4EHP, Paps CG6175, CG5966, Pfrx, ed, LanA *(BG02113)*, bol, CG32529, amn, CG32556, CG8188, Crc, CG31531, Lsd-2*	*Nmdar1, Vps 33B, spict, vsg, sgl,, ttk, E2f, SF1, mam, CG15312, toc, Pk61C, bif, LanA *(BG02380, BG02469)*, CG34360*
Females				
Number of lines	31 (34.8%)	9 (10.1%)	22 (24.7%)	27 (30.4%)
Genes affected by *p[GT1] *insertions	*CG1678, tal-1A, inv, nemy, CG42708, boi, mbl, Dnr1, CG42684, Paps, CG6175, CG5966, SF1, CG30492 *(BG01990)*, trn, Smr *(BG02262)*, inx7, CG32556, CG8188, CG6301, Lsd-2*	*Trl,Osi9, fz, CG6767, CG6540, ttk, CG16708, Vha16*	*CG11226, Karl, βInt-ν, vsg, aret, sd, tmod, CG32038, l(3)neo38, bun, ade5, CG12717, CG15309, ed, NFAT, CG32529, amn, Crc, CG31531, CG14478*	*wb, msn, spict, Xrp1, CG33691, CG30492 *(BG01354)*, fs(1)h, E2f, Mer, α-Est10, CG11382, Fas3, toc, CG31145, LanA *(BG02113)*, nuf, bol, dally, jing*

Classification of significant lines according to the spatial scale of wing shape deformations for each sex. Group I: lines in which the *P*-element insertion caused general deformations; Group II: lines which showed localized deformations; Group III: lines in which the mutation caused general deformations as well as localized deformations; Group IV: lines which showed intermediate deformations. Candidate genes that could be identified are given for each group of lines in males and females.

Additionally, in order to investigate the association of specific GO terms with any particular group (I-IV), all significant lines were ranked according to their effects on wing shape in comparison to the control line for each sex independently. For both sexes, lines belonging to group III showed the strongest wing shape variation compared to the control. In females, genes belonging to this group were enriched in signal transduction and cell communication. However, in males, no functional association was detected.

### Variance and correlation analyses of landmarks

**Figure 4:**
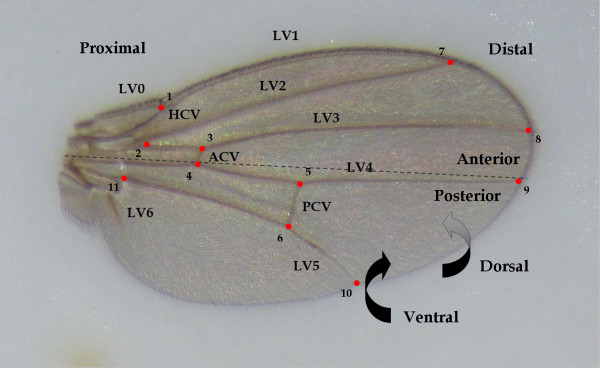
***Drosophila*'s wing morphology**. Ventral view of left wing and landmark positioning. LV: longitudinal vein, HCV: humeral crossvein, ACV: anterior crossvein, PCV: posterior crossvein. The proximal, distal, anterior-posterior and dorsal-ventral axes are shown.

The mean Procrustes coordinates of the significant lines were estimated for each group (I-IV) and sex separately to study the distribution of the values corresponding to different lines around each landmark. According to our results (Table 3), no landmark had the largest variance in both X and Y dimensions nor did any landmark have the smallest variance in both X and Y dimensions. We detected that variances of Y3, Y4 and Y5 were among the five lowest values considering all groups and both sexes (Table 3). Contrarily, variance associated to X5 was one of the largest values in all cases except for males of group IV while variances of X6, X7 and X10 were among the five largest values in six out of eight cases (Table 3). These results suggest that the lowest variance was seen for the Y component of proximal landmarks whereas the largest variance was seen for the X component of distal landmarks (Figure 4).

**Table 3:** Principal results of the variance analyses of individual landmarks.

	Females				Males			
**Coordinate**	**I**	**II**	**III**	**IV**	**I**	**II**	**III**	**IV**

X1	61	73	166	60	103	19	80	**88**
Y1	76	31	36	33	58	22	60	72
X2	**176**	36	148	**71**	70	38	91	**88**
Y2	20	22	24	7	19	5	19	24
X3	68	**84**	**240**	52	**171**	**60**	**108**	46
Y3	5	4	9	8	13	10	12	8
X4	57	63	**212**	46	**138**	49	89	25
Y4	7	9	15	13	6	7	9	21
X5	**173**	**157**	**336**	**93**	**214**	**76**	**276**	80
Y5	30	8	16	16	20	9	18	15
X6	**142**	**329**	**676**	**88**	**189**	47	**369**	79
Y6	64	42	101	26	95	25	96	29
X7	**347**	**97**	165	**189**	91	**51**	**150**	**173**
Y7	105	**199**	160	36	52	**60**	74	70
X8	109	29	119	34	56	11	79	**87**
Y8	42	20	48	25	34	19	33	22
X9	85	9	53	29	47	18	62	22
Y9	33	14	51	29	26	15	37	37
X10	**205**	57	**233**	**74**	**120**	22	**148**	**197**
Y10	126	43	126	49	50	**50**	58	65
X11	56	82	91	31	65	9	77	32
Y11	42	14	60	32	22	29	30	40

Variance of values of each coordinate (X and Y) corresponding to each landmark (1 to 11) estimated using the mean of the values of each line for each group (I-IV) and sex separately. The five largest values are bold-faced and the five lowest values are underlined.

We also evaluated the significance of the correlation between X and Y values corresponding to each landmark in order to determine if each landmark can move in all possible directions. The correlation was significant in 12 and 5 cases (out of 44 analyses per sex) in males and females respectively; being positive in 5 cases in males and 4 cases in females (Table 4). Those significant correlations were distributed among groups I, II and III in both sexes and only 3 of them showed a similar pattern between sexes: X and Y values corresponding to landmarks 3 and 9 showed a positive correlation in groups I and II respectively; and coordinates related to landmark 6 presented a negative correlation in group III (Table 4). These results imply that the intersection between longitudinal vein 3 and the anterior cross-vein as well as the intersection between longitudinal vein 4 and the margin of the wing would move frequently between posterior-proximal to anterior-distal positions in both sexes (Figure 4). These results also suggest that the intersection between longitudinal vein 5 and the posterior cross-vein would move mostly between posterior-distal to anterior-proximal positions in both sexes (Figure 4). The percentage of total variance explained by the correlation (*r*^*2 *^× 100) ranged from 14% (landmark 3 in males of group III and females of group I) to 55% (landmark 3 in males of group I and landmark 10 in females of group II; Table 4). Therefore, deformations related to each landmark occurred mostly in all directions (i.e. they showed an isometric pattern), with a few exceptions corresponding to the cases mentioned before which might implicate the existence of preferred shape changes along particular directions.

**Table 4:** Principal results of the correlation analyses of individual landmarks.

	Males				Females			
**Landmark**	**I**	**II**	**III**	**IV**	**I**	**II**	**III**	**IV**

1	-0.22	0.08	-0.19	0.17	0.24	0.28	0.16	-0.06
2	***-0.52***	0.38	***-0.46***	0.25	***0.66***	0.21	0.05	-0.05
3	***0.74***	0.08	***0.38***	0.10	***0.38***	0.35	0.36	0.18
4	-0.28	***-0.66***	-0.31	0.24	0.13	-0.58	-0.28	0.03
5	-0.15	0.39	0.14	0.15	0.15	0.25	0.06	0.13
6	***-0.45***	0.07	***-0.56***	0.09	0.23	-0.54	***-0.62***	-0.04
7	***-0.47***	-0.19	-0.22	0.03	-0.27	0.35	-0.17	-0.30
8	-0.12	-0.20	0.16	0.04	-0.02	0.05	0.14	-0.05
9	0.28	***0.62***	0.05	0.23	-0.09	***0.73***	0.06	-0.04
10	***0.60***	0.06	***0.40***	0.03	0.20	***0.74***	0.31	0.11
11	-0.21	0.37	***-0.40***	0.15	0.24	-0.61	-0.33	-0.17

Principal results of the correlation analyses between X and Y coordinates of each landmark. A correlation analysis was performed between each pair of coordinates within males and females separately. The mean of the values of each coordinate corresponding to each line for each group (I-IV) was used in the analysis. *r *value for each correlation analysis is shown. Significant values (p < 0.05) are bold-faced and italicized.

### Visualization of wing shape deformations

**Figure 5:**
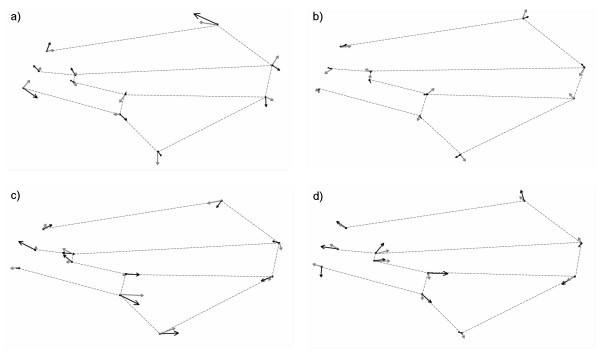
**Wing shape deformations for males and females**. Wing shape deformations in males (black arrows) and females (grey arrows) associated with the mutation of candidate genes *invected *(BG00846, a), *frizzled *(BG01047, b), *scalloped *(BG01633, c) and *CG31531 *(BG02612, d). Arrows indicate the magnitude and direction of landmark displacement with respect to the corresponding control line. Arrow size has been magnified three times to show wing shape changes more clearly.

**Figure 6:**
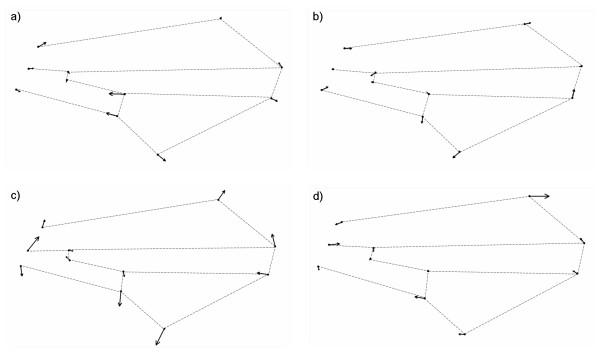
**Wing shape deformations for males or females**. Wing shape deformations specific to males (a, c) and females (b, d). a) *l(3)82Fd *(BG01597), b) *CG6767 *(BG01218), c) *LamininA *(BG02469) and d) *jing *(BG02314). Arrows indicate the magnitude and direction of landmark displacement with respect to the corresponding control line. Arrow size has been magnified three times to show wing shape changes more clearly.

One line belonging to each one of the groups mentioned before was selected for each sex separately to show examples of wing shape deformations caused by *P*-element insertions in different candidate genes. In general, these lines were among those which showed the largest wing shape changes compared to the control in their respective group in at least one sex. Therefore, in some of these cases wing deformations are shown for both sexes (Figure 5), while in other cases they are shown only for a single sex (Figure 6).

The *P*-element insertion in *inv *(BG00846) affected practically the whole wing (Figure 5a). Landmark displacements were similar in magnitude and they were generally intermediate (Group I). However, some landmarks showed larger displacements, especially in males (7 and 11; Figure 5a). Finally, the direction of changes was very different between sexes (Figure 5a). Mutation of *fz *(BG01047) affected some parts of the wing more than others although landmark displacements were relatively small (Figure 5b). In this gene that belongs to group II, landmarks 1, 7 and 10 showed larger displacements in males whereas landmarks 1, 2, 5, 7 and 8 revealed that pattern in females (Figure 5b). The direction of changes was also dissimilar between males and females (Figure 5b). The *P*-element insertion in *sd *(BG01633), which corresponds to group III, affected the entire wing (Figure 5c). Even though landmark displacements were not similar in magnitude, they were generally large (Figure 5c). In particular, landmarks 2, 6 and 10 showed the largest displacements in males whereas landmarks 6, 7 and 10 showed the largest displacements in females (Figure 5c). Finally, the direction of changes seemed to be more similar between sexes than in the previous cases (Figure 5c). The mutation affecting the group III gene *CG31531 *(BG02612) also deformed the whole wing with landmark displacements very different in magnitude (Figure 5d). In particular, landmarks 2, 5 and 9 showed the largest displacements in males whereas landmarks 3 and 4 revealed that pattern in females (Figure 5d). The direction of changes was evidently similar between sexes only with respect to some of the landmarks (Figure 5d).

The *P*-element insertion in *l(3)82Fd *(BG01597) affected some parts of the wing remarkably more than others in males (Figure 6a). For this gene, that belongs to group I, landmarks 1, 5, 6 and 10 showed the largest displacements with the most prominent changes in the intermediate region of the wing (Figure 6a). Mutation of *CG6767 *(BG01218, Group II) affected some parts of the wing more than others in females, although landmark displacements were relatively small (Figure 6b). Displacements of landmarks 1, 3, 6, 7, 9, 10 and 11 were remarkably larger than the others (Figure 6b). The next two examples correspond to genes that belong to group IV. The *P*-element insertion affecting *LanA *(BG02469) caused modifications in the entire wing in males (Figure 6c). Even though landmark displacements were not similar in magnitude; they were generally large (Figure 6c). Landmarks 2, 6 and 10 showed the largest displacements whereas landmarks 7, 8, 9 and 11 moved slightly less (Figure 6c). Finally, the mutation affecting *jing *(BG02314) caused significant modifications in some parts of the wing in females (Figure 6d). Landmarks 2, 6 and especially landmark 7 showed the largest displacements (Figure 6d).

## Discussion

In recent years, numerous studies have been performed to study the genetic basis of wing form in *D. melanogaster *[6,12,22-32,35].Mutagenesis by *P*-element insertion has been recently used to analyze wing shape variation in this species [12,32]. The principal difference between those studies and this one is that we used a larger number of mutants. Our results represent a substantial contribution to wing shape genetics because there seems to be little overlap between the candidate genes identified in this study and those identified in previous studies. In fact, for most of the candidate genes identified, this is apparently the first record relating them to wing shape variation.

### Analyses of divergent lines for wing size and shape

Although the number of genes affected by *P*-element insertions in our array of lines only represents 1 to 1.5% of all the genes in the *Drosophila *genome, more than 63% of these induced mutations affected wing shape in one or both sexes. This result suggests that a large number of genes contribute to the expression of this trait which is in line with previous estimates [12,22,24,26-32]. However, an interesting observation was that 67% of the lines showed wing shape changes only in one sex. Thus, the genetic basis of wing shape seems largely sex specific, which is contrary to previous findings that showed a sex independent expression of the genes [22,31]. In turn, these results have apparently led some authors to analyze only one sex in their studies [26,28,29,32]. Other authors found differences between sexes for wing shape, [24] but used only one of them to search for QTLs for that trait in a subsequent work [27]. Lately, Dworkin and Gibson [12] analyzed both sexes utilizing the same methodology used in this study and found significant differences between sexes but they did not stress that result presumably because it was not their primary objective. Our results, that have been obtained analyzing a number of lines/genes that was larger than in previous studies, show that the genetic basis of wing shape depends largely on the sex considered. Several microarray studies have indicated that thousands of genes distributed throughout the genome contribute to sexual dimorphism in *D. melanogaster *[36,37]. Further, several analyses of gene expression have investigated the strength and molecular signal of sex-specific evolutionary forces [37-43]. However, these studies were performed using adult flies whereas studies of expression patterns of specific tissues (e.g. wing imaginal discs in third instar wandering larvae) have not discriminated between sexes [30,44,45]. Therefore, it would be very helpful for future investigations to analyze the expression patterns of wing imaginal discs in males and females separately. In general, the results of the studies mentioned previously indicate that sex-biased genes show a nonrandom genomic distribution which is consistent with the hypothesis that past intralocus sexual conflict at many loci has been at least partially resolved through the evolution of sex-specific levels of gene expression (reviewed in [46]). Our data indicate that *P*-element insertions affecting wing shape mapped more frequently to the X chromosome than expected when males and females were analyzed together (χ^2^1 = 6.133, p = 0.013). However, distribution of candidate genes between the X chromosome and the autosomes did not depart from the expected distribution (according to chromosomes' approximate size) when the sexes were analyzed separately (χ^2^1 = 0.144, p = 0.704 for males; χ^2^1 = 0.075, p = 0.784 for females). Even though these results are apparently contrary to previous studies [37,41,47], other investigations have indicated that the nonrandom distribution of sex-biased genes are due to chromosome inactivation and dosage compensation rather than sexually antagonistic selection [46]. Moreover, some evidence suggests that the autosomes play an important role in the evolution of sexual dimorphism [36,46]. Finally, to our knowledge, only two recent investigations have analyzed sexual dimorphism of wing shape in *Drosophila *[48,49]. Those studies suggested that wing shape is under the influence of both natural and sexual selection, reinforcing the idea that the evolution and development of wing morphology should be studied in both sexes.

The joint analysis of wing size (trait analyzed exhaustively in [16]) and wing shape indicate that 79% of the lines considered showed significant differences from the control for at least one of the traits in either sex. However, only 19% of the lines showed those differences for both traits in one or both sexes. The percentage of lines in which the *P*-element insertions affected only wing shape is almost three times the percentage in which the same insertions affected only wing size. Further, the results of the frequency analyses suggest that these traits show an independent behaviour with respect to the genes involved in their expression. These results indicate that the genetic basis of wing shape and wing size are largely independent of each other which is consistent with previous observations [12,22,24,26-32]. Finally, only a third of the mutations caused similar effects in wing form in both sexes (changing wing size and/or shape). This value is similar to that associated with males but it is larger than the value corresponding to female specific lines. These results suggest that the expression of wing size and wing shape involve a large part of the genome although many of the genes are likely trait and sex specific. Our results highlight that the genetic architecture of wing size differs from that of wing shape, as has been observed by other authors in natural populations of *D. melanogaster *[23].

### Analyses of wing deformations and associated genes

The results of the correlation analyses showed that deformations related to each landmark generally occurred in all directions which indicates the general absence of preferred (or, on the contrary, avoided) shape changes along any particular direction. However, analyses of variance of the values related to each landmark revealed that the intersection between veins showed the smallest displacements in the proximal region of the wing. This suggests that development of the proximal region is canalized compared to other parts of the wing which might be due to its proximity to the intersection of the wing and the body.

The analyses performed to classify the significant lines according to the spatial scale of wing shape deformations revealed that mutations causing global changes were more common than expected in both sexes. This suggests that a large proportion of the genes identified are involved in early wing morphogenesis (the period in which dorsal-ventral and anterior-posterior axes are established and the positions of the future veins are determined) [3]. These analyses also revealed that mutations causing localized wing deformations were less common than expected in both sexes. This implies that only a small number of the genes identified are involved in wing formation during late development (the stage in which refinement of venation pattern occurs) [3]. Therefore, global wing shape changes might be due to altered vein positioning whereas localized deformations might be due to local changes in growth which may not significantly affect other regions of the wing. Future investigations could deal with this issue by analyzing the expression patterns of the respective tissue during different stages of pupal development.

We observed little overlap between the candidate genes identified in this study and those found in previous studies. In fact, for most of our candidate genes, this is the first record relating them to wing shape variation (See additional file 1).Among the other genes which have been previously associated with wing development and/or vein patterning are a few that should be analysed in greater detail. *invected *(*inv*) encodes a transcription factor involved in the determination of the anterior-posterior identity of the wing [50,51]. This might explain the pronounced effect that its mutation produced on wing shape in both sexes. Moreover, the global effects observed are consistent with early expression during wing organogenesis. Interestingly, many fitness related traits, including wing size [16] and developmental time [19,52] are also affected by this mutant.

*scalloped *(*sd*) has been largely related to wing morphogenesis as part of the Wg signaling pathway (for example, [53]). Scalloped and Vestigial (Vg) constitute a dimeric functional transcription factor in which Vg provides the transcription activator function while Sd binds DNA [54,55]. This might explain the effect that a mutation of this gene (BG01633) had on wing shape in both sexes, which also caused a significant increase in wing size in males and females [16]. However, it must be mentioned that another mutation of the same gene (BG02605), only caused an increase in wing size in males [16]. The mutations might have affected certain sites of *sd *in such a way that the resultant product (if there is any) can not bind adequately to DNA and/or Vg.

*Laminin A *(*LanA*) encodes one of the chains that forms a heterotrimer belonging to the extracellular matrix that binds to position-specific integrins [56]. Adhesion between the two surfaces of the wing fails when integrin function is reduced [56,57]. Thus, mutation of *LanA *might interfere with the normal function of these integrins causing developmental modifications. As a result, this may produce dissimilar wing form changes, as was seen in different mutant lines (BG01662, BG02113, BG02380 and BG02469; [16]). Again, the mutations might have affected certain sites of *LanA *in a way such that the resultant product can not bind adequately to the respective integrins. Further investigation of *sd *and *LanA *(e.g. expression analyses and molecular studies) may help to elucidate the cause of the differential phenotypic effects associated to different mutations of the same gene.

The functional analysis of the 94 candidate genes that have been identified for wing shape revealed that a large proportion of the annotated genes are involved in development and/or metabolic processes, which is in line with previous observations [32]. Furthermore, the results showed that the products of most of them are located in intracellular organelles and present protein binding activity. The functional enrichment analysis revealed that some GO terms were over-represented in our list of candidate genes compared to *D. melanogaster*'s genome. Some of the terms are suggestive because they are very similar to those that have already been related to organ development (e.g., [58]). For instance, in pupae, wing cells reconstitute their contacts generating a highly ordered hexagonal cover by a mechanism implicating several components of the planar cell polarity pathway [59]. Such a process is influenced by cell surface mechanics and by local cell division rates [60]. Tissue growth is also affected by cell intercalation, a mechanism that has been observed during the evagination of the pupal imaginal wing disc [61], in which cells change position by reconstructing their adhesive contacts [60]. These examples illustrate processes in which some candidate genes related to GO terms such as regulation of cell adhesion, regulation of cell motility and plasma membrane might be involved. Moreover, in females, mutations showing the strongest effects on wing shape variation were associated with the GO terms of cell communication and signal transduction in agreement with the idea that an organ's shape results from local cell interactions rather than from cells' response to global controls [62].

## Conclusions

Our results support the idea that the genetic architecture of wing shape is complex with many different genes contributing to the trait in a sexually dimorphic manner. Its largely polygenic basis, which is relatively independent from that corresponding to wing size, is composed of genes generally involved in development and/or metabolic functions, especially those related to the regulation of different cellular processes such as motility, adhesion, communication and signal transduction. Our results suggest that understanding the genetic basis of wing shape requires merging the regulation of vein patterning by signalling pathways with processes that occur during wing development at the cellular level. Further studies might help to elucidate the role of our candidate genes in wing patterning by relating them to known or novel signaling pathways. This will help to improve our knowledge of *Drosophila *wing development.

## Methods

### Drosophila stocks

We used 191 independent lines, homozygous for a single *P[GT1]*-element insertion, constructed in a coisogenic *Canton-S *background [13] to identify candidate genes affecting wing shape. These lines have been previously used to study different body size related traits including wing size [16] and were kindly provided by Trudy Mackay (North Carolina State University, Raleigh, NC, USA).

### Experimental design

*P*-element insertion lines were randomly distributed in nine batches in which 20-25 lines were simultaneously assessed. To account for environmental variation in wing shape between batches, 4-8 replicate vials of the control strain (a co-isogenic line lacking the *P*-element insertion) were run in parallel with each batch. For each line, 300 pairs of sexually mature flies were placed in oviposition chambers for 8 hours. Eggs were allowed to hatch and batches of 30 first-instar larvae were transferred to culture vials containing a standard cornmeal-agar-molasses medium (4 replicates per line). Larvae were raised at 25 ± 1°C and 60-70% humidity with a 12:12 hour light:dark photoperiod until adult emergence.

### Wing measurements

Four flies of each sex from each vial were randomly chosen and the wings of each individual were removed and mounted on slides. Images were captured using a binocular microscope (10×) and an attached digital camera connected to a computer. For the estimation of wing shape, 11 landmarks were digitized on the ventral face of the left wing of each fly (Figure 4) using tpsDig [63]. Shape variation was investigated using the Procrustes generalized least squares procedure [64], which allows for the superimposition of all the wings. This eliminates the variation in size, position and orientation of the wings and allows for the examination of the differences in the position of the landmarks. This procedure generated 22 new Procrustes coordinates and eliminated four degrees of freedom, resulting in 18 shape-space dimensions [65]. Shape variables generated afterwards, known as partial warps, indicate the partial contributions of hierarchically scaled vectors spanning a linear shape-space. Subsequently, principal components analysis of the partial warps scores matrix was conducted to obtain 18 new shape variables called relative warps (RWs; [65]). RWs scores were estimated employing different values of α [66] using tpsRelw [67]. A value of α = 0 gives equal weight to all partial warp scores, regardless of their spatial scale; a value of α = 1 gives greater weight to partial warp scores related to larger spatial scales, involving general deformations; and a value of a α = -1 gives greater weight to partial warp scores corresponding to smaller spatial scales, concerning local changes.

### Statistical and morphometrical analyses

#### Identification of divergent mutant lines for wing shape

Multivariate analyses of variance (MANOVAs) were performed to detect significant differences in wing shape between mutant lines and a control using the RWs scores corresponding to a value of α = 0 (see above). A MANOVA was conducted for each *P*-insert line and the respective control line in males and females separately. Since the values of each control were used as many times as the number of lines of the respective batch, Bonferroni's correction for multiple tests was applied to the analyses corresponding to each batch. Lines that exhibited significant differences relative to the control were considered to have an insertion in (or near) a candidate gene for wing shape.

#### Simultaneous analysis of both components of wing form

Data corresponding to wing size were taken from our previous work in which different body size related traits have been analyzed [16]. The number of cases in which *P*-element insertions affected wing size and/or wing shape was subjected to a frequency analysis in each sex separately.

#### Variance and correlation analyses of individual landmarks

We obtained the Procrustes coordinates (see above) corresponding to each group and sex and estimated the mean value for each one of them (i.e. 11 X-coordinates and 11 Y-coordinates) for every line. The variance of the mean values corresponding to each coordinate was estimated. These estimates allowed us to investigate whether or not the variance around each landmark varied across the wing in order to assess the extent to which each region might change. Also, the correlation between the mean values of X and Y corresponding to each landmark was analyzed. These analyses permitted us to study whether deformations related to each landmark occurred in all possible directions (showing an isometric pattern) or happened predominantly in certain ways. An isometric dispersion of lines around a particular landmark might function as a null hypothesis as there is no a priori favoured direction for shape changes. On the contrary, a significant correlation between the X and Y values of a landmark would indicate the existence of preferred shape changes along a particular direction (i.e. shape changes in different directions would not be equally probable).

All the statistical analyses mentioned above were performed using the STATISTICA software package [68].

#### Gene identification and functional analysis

To identify the transposon-tagged candidate genes for wing shape, nucleotide sequences flanking the *P*-element insertion were aligned with corresponding sequences of the reference sequence of the *D. melanogaster *genome. Homology searches were performed against release 5 of the published *D. melanogaster *genomic sequence [69]. Candidate genes were distributed into different gene ontology (GO) terms corresponding to the categories "biological process" and "molecular function" according to their annotations [34]. Furthermore, this frequency distribution, as well as that corresponding to all of the lines, was compared to the frequency distribution of all of the genes of *D. melanogaster *provided by FlyBase [69]. These comparisons allowed us to assess whether our list of candidate genes for wing shape is enriched with genes belonging to certain GO categories. All of these analyses were performed automatically with the aid of the programme FatiGO [70]. Finally, we performed another gene set enrichment analysis employing the program FatiScan [70] in order to study the association of specific GO terms to any particular group (I-IV). In this case, all significant lines were ranked according to their effects on wing shape in comparison to the control line, for each sex separately, to look for blocks of functionally related genes with similar phenotypic effects across the list. Both programmes belong to the Babelomics suite of bioinformatic tools which is available online [71]. They both distribute the genes among the terms corresponding to each category in a non-exclusive manner (that is, a given gene might be associated to more than one GO term). These programmes correct *p*-values for multiple testing by the widely accepted FDR method [72].

#### Classification of significant lines according to the spatial scale of wing shape deformations

Dunnett contrasts (control versus each line) were performed for each batch and sex using the scores of the first three RWs (those which explained more than 50% of total shape variance) corresponding to a value of α = -1 and α = 1 (see above) separately. According to the results of these a priori contrasts, significant lines were distributed among the following groups in each sex separately: I) lines which showed significant differences with respect to the control for any of the first three RWs estimated with a value of α = 1; II) lines which showed those differences for any of the first three RWs estimated with a value of α = -1; III) lines which showed those differences for any of the first three RWs estimated with a value of α = 1 as well as with α = -1; IV) lines which did not show those differences for any of the first three RWs estimated with a value of α = 1 nor with α = -1 (but otherwise with significant differences with respect to the control). Consequently, lines belonging to group I were those which showed deformations that involved almost the whole wing; lines belonging to group II were those which showed localized deformations; lines belonging to group III were those which showed deformations at both extreme scales simultaneously; and lines belonging to group IV were those which showed deformations at an intermediate level. The distribution of significant lines among different groups was compared between sexes to see if certain deformations were more common in one sex than in the other. Then the number of significant lines corresponding to different groups was subjected to a frequency analysis in each sex separately to determine if certain spatial scales were under- or over-represented.

#### Visualization of wing shape deformations

At least one line belonging to each one of the groups mentioned above was selected for each sex to show examples of wing shape deformations caused by *P*-element insertion in different candidate genes. Differences in wing shape between each one of these lines and the respective control line were estimated performing a thin-plate spline RWs analysis using the Procrustes coordinates of the corresponding lines [65]. This analysis allowed us to visualize differences in shape between the mutant and the control lines as changes in the deformation grid of the control wing. Shape changes of each line with respect to the control were shown as vector diagrams obtained using tpsSplin [73].

## Authors' contributions

VPC conceived and designed the study, carried out the experimental crosses, egg collections, larval seeding and rearing and adult collection, took all the photographs, performed the morphological quantification, statistical and morphometrical analyses and drafted the manuscript. IMS participated in the design of the study, the morphometrical analyses and helped with the first draft of the manuscript. JM participated in the experimental crosses, egg collections, larval seeding and rearing, adult collection, genes functional analyses and helped to draft the manuscript. JJF participated in egg collections, larval seeding and rearing and adult collection, assisted in the identification of the transposon-tagged candidate genes and helped to draft the manuscript. All authors read and approved the final manuscript.

## Authors' information

JM is recipient of a post-doctorate scholarship of Consejo Nacional de Investigaciones Científicas y Técnicas (CONICET). VPC, IMS and JJF are members of Carrera del Investigador Científico of CONICET.

## Supplementary Material

Additional File 1 **Genetic information of candidate genes for wing shape**. Lines in which the *P*-element insertion affected wing shape in either sex. The candidate gene and the site of the mutation are given. Click here for file
