# Supplementary material for: Genetic basis of wing morphogenesis in Drosophila: sexual dimorphism and non-allometric effects of shape variation
Source: BMC Dev Biol. 2011 Jun 2;11:32. doi: 10.1186/1471-213X-11-32 (PMC3129315; doi:10.1186/1471-213X-11-32)
Supplement: Additional File 1 — Genetic information of candidate genes for wing shape. Lines in which the P-element insertion affected wing shape in either sex. The candidate gene and the site of the mutation are given. [file 1471-213X-11-32-S1.DOC]

|  |  |  |  |  | |
| --- | --- | --- | --- | --- | --- |
|  |  |  | Cytogenetic | Phenotypic effect | |
| Line | Candidate gene | *p[GT1]* insertion site | map | Males | Females |
|  |  |  |  |  |  |
|  |  |  |  |  |  |
| BG00177 | No significant similarity found |  |  | *** | ns |
| BG00372 | *CG1678* | 1498 bp at 3´ side | 20A1 | *** | *** |
| BG00373 | *CG11226* | 1531 bp at 5´ side | 80A4 | *** | *** |
| BG00386^m^ | *NMDA receptor 1* (*Nmdar1*) | 240p at 3´ side | 83A6-A7 | *** | ns |
| BG00429 | No sequence |  |  | *** | *** |
| BG00489 | *Osiris 9* (*Osi9*) | 253 bp at 3´ side | 83E2 | ns | *** |
| BG00524 | *tal-1A* | In gene (5´UTR) | 87F14 | *** | ** |
| BG00664 | No sequence |  |  | *** | *** |
| BG00683 | *Vacuolar protein sorting 33B (Vps 33B)* | 440 bp at 3´ side | 96E1 | * | ns |
| BG00735 | *schizo* (*siz*) */ CG10581* | 1 bp at 3´ side / 210 bp at 5´ side | 78A5 | *** | ns |
| BG00737 | *Heat shock protein 27* (*Hsp 27*) | In gene (exon) | 67B3 | ** | ns |
| BG00846^m,f^ | *invected* (*inv)*^‡^ | In gene (intron) | 47F15-F17 | *** | *** |
| BG00930 | No sequence |  |  | ns | ** |
| BG00985 | No sequence |  |  | ** | *** |
| BG00990 | *Wing blister* (*wb*)^‡^ | 515 bp at 3´ side | 35A3-A4 | ns | ** |
| BG01007 | *nemy / CG42708* | 193 bp at 3´side / 229 bp at 5´side | 49B12 | *** | *** |
| BG01010 ^m,f^ | *Karl* | In gene (exon) | 10E3 | *** | *** |
| BG01011 ^m,f^ | *misshapen* (*msn*)^‡^ | 1402 bp at 3´ side | 62E6-E7 | *** | *** |
| BG01014 | *Spichthyin* (*spict*)^‡^ | In gene (exon) | 33F3 | *** | ** |
| BG01017 | No significant similarity found |  |  | ns | ** |
| BG01018 | *forkhead box, sub-group O* (*foxo*)^‡^ | 104 bp at 3´ side | 88A5-A8 | *** | ns |
| BG01019^m^ | No significant similarity found |  |  | *** | * |
| BG01028 | *Trithorax-like* (*Trl*)^‡^ | In gene (intron) | 70F4 | *** | *** |
| BG01037 | *βν integrin* (*βInt-ν*) | In gene (3´UTR) | 39A1 | *** | *** |
|  | | | | | |
| *(continued)* | | | | | |
|  | | | | | |
|  | | | | | |
| Additional file 1. Continued | | | | | |
|  |  |  |  |  |  |
|  |  |  |  |  |  |
| BG01045 | No significant similarity found |  |  | ** | ** |
| BG01047^m^ | *frizzled* (*fz*)^‡^ | In gene (5´UTR) | 70D4-D5 | * | *** |
| BG01062 | *brother of iHog (boi)* | In gene (intron-exon) | 3A3 | *** | ** |
| BG01065 | *visgun* (*vsg*) | In gene (5´UTR) | 67C5 | *** | *** |
| BG01066 | No sequence |  |  | ** | ns |
| BG01081 | *Glutamate oxaloacetate transaminase 1* (*Got1*) | In gene (intron-exon) | 52E7-E10 | *** | ns |
| BG01127 | *Muscleblind* (*mbl*)^‡^ | In gene (5´UTR) | 54A | ** | *** |
| BG01214 | *sugarless* (*sgl*)^‡^ | In gene (5´UTR) | 65D4-D5 | ** | ns |
| BG01218 | *CG6767* | In gene (intron) | 67C4-C5 | ** | *** |
| BG01228^m,f^ | *derailed* (*drl*) | 22728 bp at 5´ side | 37C7 | ns | * |
| BG01247 | *SRY interacting protein 1* (*Sip1*) | In gene (5´UTR) | 54B6-B7 | *** | ns |
| BG01279^f^ | *Xrp1*^‡^ | In gene (intron) | 91D3-D5 | ns | * |
| BG01290 | *Btk family kinase at 29A* (*Btk29A*) | In gene (intron) | 29A1-A3 | *** | ns |
| BG01297^m,f^ | *CG33691* | 291 bp at 3´ side | 6E2 | ns | * |
| BG01314^f^ | *CG6540* | In gene (5´UTR) | 17C7 | ns | * |
| BG01354 | *CG30492* | 255 bp at 5´ side | 43E5-E7 | ** | ** |
| BG01491 | *tramtrack* (*ttk*)^‡^ | In gene (intron) | 100D1 | * | *** |
| BG01515 | *female sterile (1) homeotic (fs(1)h)* | In gene (5´UTR) | 7D3-D5 | *** | *** |
| BG01520 | *E2F transcription factor* (*E2f*)^‡^ | In gene (intron) | 93E9-F1 | * | ** |
| BG01543 | *Merlin* (*Mer*)^‡^ | In gene | 18 E1 | ** | *** |
| BG01548 | *α-Esterase-10* (*α-Est10*) | 2571 bp at 5´ side | 84D8-D9 | * | ** |
| BG01562 | *headcase* (*hdc*)^‡^ | 4750 bp at 3´ side | 99E4-F1 | *** | ns |
| BG01563 | *CG16708* | 51 bp at 3´ side | 82F11-83A1 | ns | *** |
| BG01565 | *Defense represor 1*(*Dnr1*) | In gene (intron) | 58E9-F1 | *** | *** |
| BG01566 | *arrest* (*aret*) | In gene (intron) | 33D3-D5 | *** | *** |
|  | | | | | |
| *(continued)* | | | | | |
|  | | | | | |
|  | | | | | |
| Additional file 1. Continued | | | | | |
|  |  |  |  |  |  |
|  |  |  |  |  |  |
| BG01568 | *CG42684* | In gene (intron) | 16C5-C8 | ** | * |
| BG01597 | *l(3)82Fd* | In gene (exon-intron) | 82F8-F10 | ** | ns |
| BG01600 | *chinmo* | 1244 bp at 5´ side | 22A5-A8 | *** | ns |
| BG01608^m^ | No significant similarity found |  |  | * | ns |
| BG01618 | *CG6398* | 142 bp at 3´ side | 16D5-D7 | *** | ns |
| BG01633^m,f^ | *scalloped* (*sd*)^‡^ | In gene (intron) | 13F1-F4 | *** | *** |
| BG01637 ^m,f^ | *Tropomodulin* (*tmod*) | In gene (exon-intron) | 99F7-F8 | ** | *** |
| BG01649 | *cricklet* (*clt*) | In gene (5´UTR) | 57F4 | ** | ns |
| BG01655^m^ | *CG32038* | In gene (5´UTR) | 67B7 | ns | ** |
| BG01660 | No sequence |  |  | *** | ** |
| BG01683 | *CG32572* | In gene (intron) | 15A3 | *** | ns |
| BG01689 | No significant similarity found |  |  | * | ** |
| BG01713 | *4EHP* | In gene (5´UTR) | 95E1 | *** | ns |
| BG01716 | *PAPS synthetase* (*Papss*)^‡^ | 800 bp at 3´ side | 76D1-D2 | *** | ** |
| BG01725 | *Letal (3) neo 38* (*l(3)neo38*) | 700 bp at 5´ side | 86E18 | ns | ** |
| BG01726^m^ | *CG11382* | 197 bp at 3´ side | 1E4 | ns | ** |
| BG01733^m^ | *CG6175* | 2725 bp at 5´ side | 68C1-C2 | ** | ** |
| BG01736^m^ | *CG5966* | 29 bp at 3´ side | 5D1 | *** | *** |
| BG01763 | No significant similarity found |  |  | ** | ** |
| BG01769 | No sequence |  |  | *** | ** |
| BG01784^m^ | *bunched* (*bun*)^‡^ | In gene (intron) | 33E5-E9 | ns | *** |
| BG01828 | No sequence |  |  | *** | *** |
| BG01835 | No sequence |  |  | * | *** |
| BG01839^m^ | No sequence |  |  | ns | * |
| BG01858 | No significant similarity found |  |  | *** | *** |
|  | | | | | |
| *(continued)* | | | | | |
|  | | | | | |
|  | | | | | |
| Additional file 1. Continued | | | | | |
|  |  |  |  |  |  |
|  |  |  |  |  |  |
| BG01859 | No sequence |  |  | *** | *** |
| BG01880 | *Fili*^‡^ | 104 bp at 5´ side | 58A3-A4 | * | ns |
| BG01892 | No significant similarity found |  |  | ** | * |
| BG01893 | *Splicing factor 1* (*SF1*) | In gene (5´UTR) | 90B4 | ** | *** |
| BG01902^m^ | *mastermind* (*mam*)^‡^ | In gene (intron) | 50C23-D3 | *** | ns |
| BG01949 | *Ade5* / *CG12717* | 130 / 550 bp at 5´ side | 11B16 | ns | * |
| BG01990 | *CG30492* | 358 bp at 3´ side | 43E5-E7 | ns | ** |
| BG02003 | *6-phosphofructo-2-kinase* (*Pfrx*) | In gene (intron) | 18C8 | *** | ns |
| BG02023^f^ | *Fasciclin 3* (*Fas3*) | In gene (exon-5´UTR) | 36F2-F4 | ns | *** |
| BG02058 | No sequence |  |  | ns | * |
| BG02063 | *CG15312* | In gene (5´UTR) | 9B1 | * | ns |
| BG02065^f^ | *toucan* (*toc*) | In gene (intron) | 23D1-D2 | * | * |
| BG02088 | *CG15309* | In gene (intron) | 9B4 | ns | *** |
| BG02095 | *echinoid* (*ed*)^‡^ | 2888 bp at 3´ side | 24D4-D6 | *** | *** |
| BG02106 | *CG31145* | In gene (5´UTR) | 95A4-A7 | ns | ** |
| BG02107 | No significant similarity found |  |  | * | ** |
| BG02109 | *tartan* (*trn*)^‡^ | In gene (5´UTR) | 70A1 | ** | ** |
| BG02113 | *Laminin A* (*LanA*)^‡^ | 680 bp at 5´ side | 65A8-A9 | ** | ** |
| BG02118^f^ | *Protein kinase 61C* (*Pk61C*)^‡^ | 35 bp at 3´ side | 61B1 | * | ns |
| BG02128^m,f^ | *Lethal (1) G0007* (*l(1)G0007*) | In gene (intron) | 12E3-E5 | ** | ns |
| BG02157 | *CG42268* | 46 bp at 3´ side | 67C6 | ** | ns |
| BG02173 | *NFAT*^‡^ | In gene (5´UTR) | 12A9-B2 | § | * |
| BG02175 | No significant similarity found |  |  | *** | *** |
| BG02180^f^ | *nuclear fallout* (*nuf*)^‡^ | In gene (exon-intron) | 70D3-D4 | ns | ** |
| BG02192^m^ | *boule (bol)*^‡^ | In gene (intron) | 66F5-67A1 | ** | *** |
|  | | | | | |
| *(continued)* | | | | | |
|  | | | | | |
|  | | | | | |
| Additional file 1. Continued | | | | | |
|  |  |  |  |  |  |
|  |  |  |  |  |  |
| BG02199^m,f^ | *Division abnormally delayed (dally)*^‡^ | In gene (5´UTR) | 66E1-E3 | * | ** |
| BG02219^m^ | *Smrter (Smr)* | 375 bp at 3´ side | 11B10-B14 | ** | ns |
| BG02241^m,f^ | *bifocal* (*bif)* | In gene (exon) | 10D4-D5 | * | ns |
| BG02262^m,f^ | *Smrter (Smr)* | In gene (intron) | 11B10-B14 | ** | *** |
| BG02286^m,f^ | *CG32529 / amnesiac* (*amn*)^‡^ | In genes (intron / exon) | 18F4-19A2 | *** | *** |
| BG02292 | No significant similarity found |  |  | *** | ** |
| BG02314 | *jing*^‡^ | 289 bp at 3´ side | 42C1-B2 | ns | ** |
| BG02359^m^ | *innexin 7* (*inx7*) | In gene (intron) | 6E4 | ns | * |
| BG02380^m^ | *Laminin A* (*LanA*)^‡^ | 151 bp at 5´ side | 65A8-A9 | ** | ns |
| BG02439 | *CG32556 / CG8188* | 300 bp at 5´ side / 950 bp at 3´ side | 16C1 | *** | * |
| BG02462 | *CG34460* | 1214 bp at 5´ side | 53D11 | *** | *** |
| BG02469^m,f^ | *Laminin A* (*LanA*)^‡^ | 170 bp at 5´ side | 65A8-A9 | *** | ns |
| BG02480 | No sequence |  |  | *** | *** |
| BG02520 | *Vacuolar H+ ATPase 16kD subunit* (*Vha16*) | In gene (intron) | 42B2 | ns | ** |
| BG02524 | *CG34360* | 28 bp at 5´ side | 85F1-F5 | *** | ns |
| BG02546^m^ | No significant similarity found |  |  | ns | ** |
| BG02563 | *Capricious* (*caps*)^‡^ | In gene (5´UTR) | 70A3-A4 | *** | ns |
| BG02566 | *Calreticulin (Crc)* | In gene (5´UTR) | 85E1 | ** | *** |
| BG02612 | *CG31531* | 2 bp at 5´ side | 82D1-D2 | *** | *** |
| BG02631 | No significant similarity found |  |  | ns | * |
| BG02690^f^ | *CG14478* | In gene (intron) | 54B16 | *** | *** |
| BG02830^m,f^ | *Lipid storage droplet-2* (*Lsd-2)* | 61 bp at 5´ side | 13A8-A9 | *** | *** |
|  |  |  |  |  |  |

^m^ Significant line for wing size (WS) in males; ^f^ Significant line for WS in females (see [16] for more details). ^‡^ Gene related to wing development according to Flybase [69]. ns: not significant; § p < 0.03; * p < 0.01; ** p < 0.001; *** p < 0.0001. ns lines for wing shape but significant for WS in either sex: BG00767 (*Guanine nucleotide exchange factor GEF64C*), BG01329 (*CG4400*), BG01361 (*CG6854*), BG01385 (No sequence), BG01433 (*Rapgap1*), BG01538 (No sequence), BG01573 (*forkhead box, sub-group O*), BG01607 (*Gliotactin*), BG01613 (*CG12161*), BG01662 (*Laminin A*), BG01709 (*kermit*), BG01717 (No significant similarity found), BG01912 (pxb), BG02019 (*CG9171*), BG02034 (*lilliputian*), BG02056 (*longitudinals lacking*), BG02081 (*Rtnl1*), BG02108 (*Doughnut on 2 / CG13086*), BG02131 (*CG31886*), BG02210 (*lamina ancestor*), BG02239 (*CG11550*), BG02306 (*CG10641*), BG02398 (*fog*), BG02412 (*CG8062*), BG02435 (*Tollo*), BG02441 (*CG32781*), BG02601 (*CG14782*), BG02605 (*scalloped*), BG02727 (*escargot*). ns lines for both wing traits and sexes: BG00369 (*CG13334/CG13333*), BG00528 (*Osiris 9*), BG00992 (*CG17574*), BG01009 (*CG17025*), BG01257 (*jing*), BG01339 (*cricklet*), BG01380 (*Oseg4 / draper*), BG01412 (No sequence), BG01488 (*musashi*), BG01498 (*Casein kinase Iα*), BG01556 (*CG12078*), BG01628 (*Malic enzyme*), BG01635 (No significant similarity found), BG01645 (*faint sausage*), BG01659 (*desert*), BG01672 (*CG1491*), BG01674 (*Neuroglian*), BG01705 (*CG9650*), BG01714 (*CG11940*), BG01735 (*CG13130 / big brain*), BG01780 (*CG11226*), BG01822 (*IGF-II mRNA-binding protein*), BG01898 (No sequence), BG01947 (*ld14*), BG02042 (*easily shocked*), BG02067 (*Isoleucyl-tRNA synthetase*), BG02102 (*mir-313/mir-312/mir-311*), BG02130 (*Thor*), BG02132 (No significant similarity found), BG02159 (*CG32666*), BG02240 (*bip1*), BG02327 (*pipsqueak*), BG02358 (*βν integrin*), BG02386 (*Sema-5c*), BG02391 (*neuralized*), BG02395 (*CG3600*), BG02415 (*capricious*), BG02529 (No sequence), BG02560 (*CG9674/nudC*), BG02747 (*rutabaga*), BG02823 (*scylla*).
